# Supplementary material for: Finasteride-Induced Inhibition of 5α-Reductase Type 2 Could Lead to Kidney Damage—Animal, Experimental Study
Source: Int J Environ Res Public Health. 2019 May 16;16(10):1726. doi: 10.3390/ijerph16101726 (PMC6572442; doi:10.3390/ijerph16101726)
Supplement: Supplementary file 1 [file ijerph-16-01726-s001.pdf]

International Journal of
Environmental Research and Public Health

Supplementary Figures

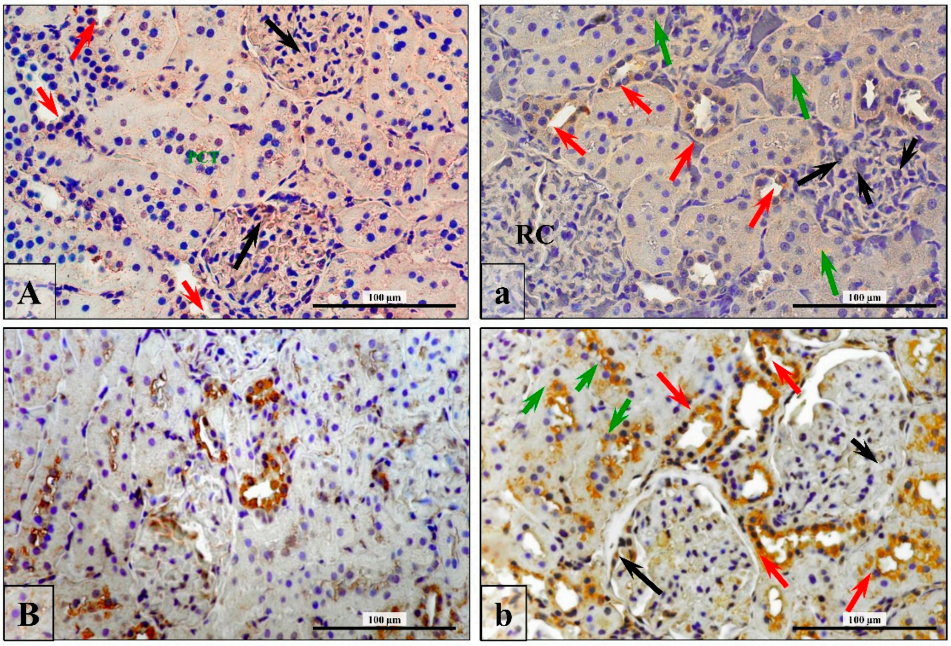

Figure 1. The representative microphotographs showing IHC reaction of occludin **(A, a)** and connexin 43 **(B, b)** expression in kidney of Control **(A, B)** and Fin **(a, b)** groups of rats. Positive result of IHC reaction are indicated by: red arrows in distal convoluted tubules (DCT), green arrows in proximal convoluted tubules (PCT), black arrows in cells within renal corpuscle (RC). Scale bars: 100 µm.

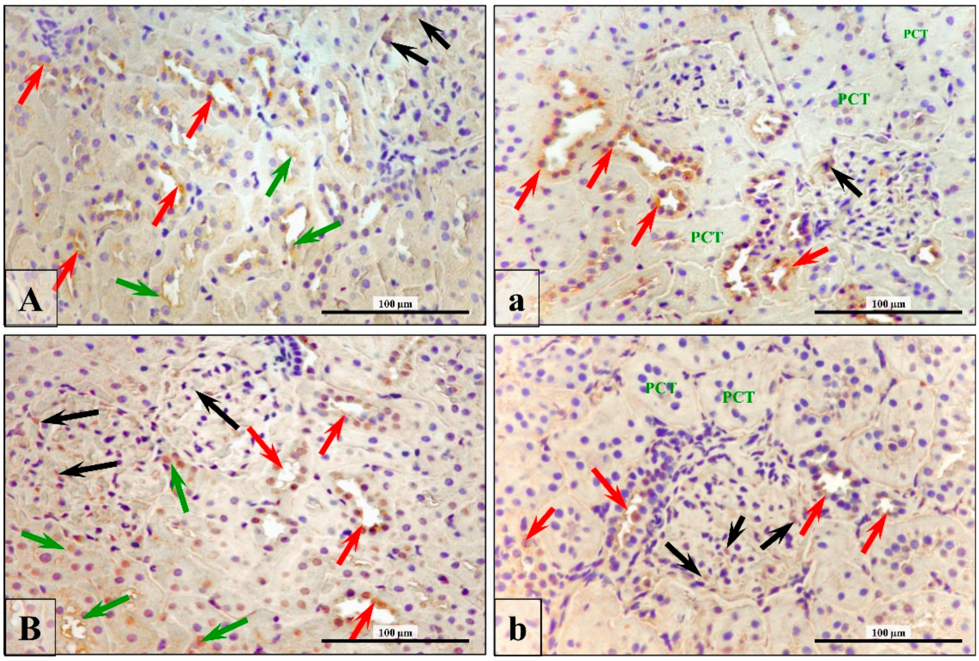

Figure 2. The representative microphotographs showing IHC reaction of E-cadherin **(A, a)** and N-cadherin **(B, b)** expression in kidney of Control **(A, B)** and Fin **(a, b)** groups of rats. Positive result of IHC reaction are indicated by: red arrows in distal convoluted tubules (DCT), green arrows in proximal convoluted tubules (PCT), black arrows in cells within renal corpuscle (RC). Scale bars: 100 µm.

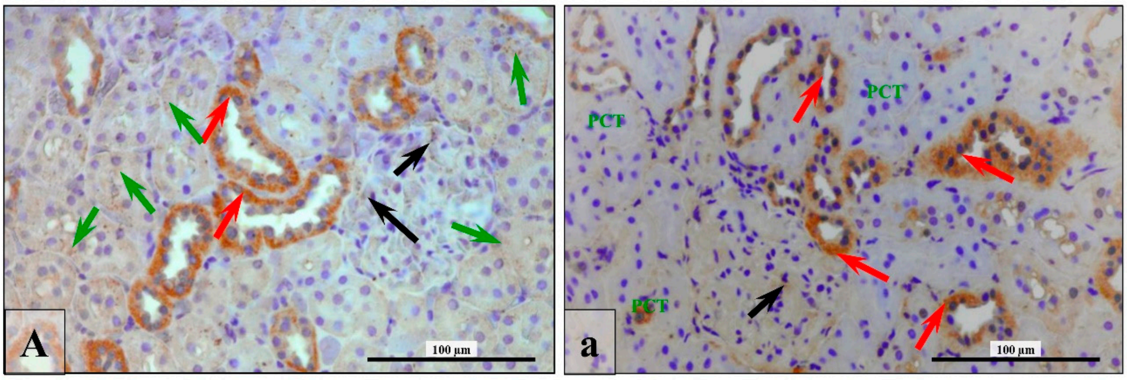

Figure 3. The representative microphotographs showing IHC reaction of β-catenin **(A, a)** in kidney of Control **(A)** and Fin **(a)** groups of rats. Positive result of IHC reaction are indicated by: red arrows in distal convoluted tubules (DCT), green arrows in proximal convoluted tubules (PCT), black arrows in cells within renal corpuscle (RC). Scale bars: 100 µm.
